# Osthole inhibits histamine-dependent itch via modulating TRPV1 activity

**DOI:** 10.1038/srep25657

**Published:** 2016-05-10

**Authors:** Niu-Niu Yang, Hao Shi, Guang Yu, Chang-Ming Wang, Chan Zhu, Yan Yang, Xiao-Lin Yuan, Min Tang, Zhong-li Wang, Tana Gegen, Qian He, Kehua Tang, Lei Lan, Guan-Yi Wu, Zong-Xiang Tang

**Affiliations:** 1 College of Basic Medicine, Nanjing University of Chinese Medicine, 138 XianLin Road, Nanjing 210023, China; 2 Jishou University, Jishou, 410023, China; 3 College of Life Science, Nanjing Normal University, Nanjing, 210046, China; 4 College of Basic Medicine, Guangxi University of Chinese Medicine, 13 WuHe Road, Nanning 530299, China; 5 The Jiangsu Collaborative Innovation Center of Traditional Chinese Medicine (TCM) Prevention and Treatment of Tumor, China

## Abstract

Osthole, an active coumarin isolated from *Cnidium monnieri* (L.) Cusson, has long been used in China as an antipruritic herbal medicine; however, the antipruitic mechanism of osthole is unknown. We studied the molecular mechanism of osthole in histamine-dependent itch by behavioral test, Ca^2+^ imaging, and electrophysiological experiments. First, osthole clearly remitted the scratching behaviors of mice induced with histamine, HTMT, and VUF8430. Second, in cultured dorsal root ganglion (DRG) neurons, osthole showed a dose-dependent inhibitory effect to histamine. On the same neurons, osthole also decreased the response to capsaicin and histamine. In further tests, the capsaicin-induced inward currents were inhibited by osthole. These results revealed that osthole inhibited histamine-dependent itch by modulating TRPV1 activity. This study will be helpful in understanding how osthole exerts anti-pruritus effects and suggests that osthole may be a useful treatment medicine for histamine-dependent itch.

Itch (pruritus) is an unpleasant cutaneous sensation^1^, and is also a symptom of many common diseases, including atopic dermatitis, thyroid diseases, diabetes mellitus, chronic renal failure, and cholestatic liver diseases^2^. People with chronic pruritus experience decreased quality of life due to sleeplessness, anxiety, depression, and embarrassment^3^. Histamine—one of the best known pruritogens—is the mediator in several conditions such as urticaria, insect bite reactions, cutaneous mastocytosis, and drug rashes^4^. Itch is often classified into histamine-dependent and histamine-independent itch. Histamine receptors are members of the G protein-coupled receptors (GPCR). Four subtypes of histamine receptors (H1–H4) have been identified^5^. Several studies found that histamine H1 and H4 receptors play a critical role in histamine-induced itch^6^. Histamine H3 receptor, as a presynaptic auto- and heteroreceptor, regulates histamine synthesis and release in the central and peripheral nervous system^7^; however, the involvement of histamine H2 receptor in histamine-dependent itch is not convincing^6^.

TRPV1—a nonselective cation channel stimulated by capsaicin, heat, and H^+^—has been implicated in mediation of pain and itch^8^,^9^. The TRPV1 channel is modulated by GPCR signalings^10^. Most pruritogens can activate GPCRs and trigger itch by activating the TRP channels, including TRPV1^9^. A growing body of evidence indicates that H1R is coupled with G_q_/G_11_ to active phospholipase Cβ3 (PLCβ3), resulting in the increase of intracellular Ca^2+^ in DRG neurons via TRPV1^11^. Histamine also activates TRPV1 via the PLA2/LO pathway, leading to the excitation of sensory neurons to induce itch^12^. In addition, *TRPV1*^−/−^ mice showed significantly attenuated scratching behavior after injection of trypsin^13^. These findings suggest that TRPV1 plays a critical role in histamine-dependent itch, especially in H1 receptor-mediated itch.

*Cnidii monnieri fructus* (dried fruit of *Cnidium monnieri* [L.] Cusson), as an herbal medicine, functions in anti-allergic, anti-dermatophytic, anti-cancer, killing parasites, and in anti-itch^14^. *Cnidium monnieri fructus* has been used for centuries in traditional Chinese medicine to treat various diseases such as sexual dysfunction, asthma, osteoporosis, and skin ailments^15^. The main constituents of *Cnidium monnieri* are coumarins, such as osthole, imperatorin, bergapten, isopimpinellin, and xanthotoxin, which have various biological activities^16^. It has been reported that osthole (7-methoxy-8 –isopentenoxycoumarin, Fig. 1) has anti-inflammatory, anti-osteoporotic, anti-tumor, and estrogen-like effects^17^,^18^,^19^,^20^,^21^. Osthole also has an antipruritic effect in allergic model animals^22^. Matsuda, H. *et al.* reported that ethanol extract of *Cnidii Monnieri Fructus* including osthole showed an inhibitory effect on compound 48/80-induced scratching behavior^23^. The precise role of osthole in the histamine-dependent itch, however, is unclear and the molecular mechanism of its anti-pruritic effect is underappreciated.

In the current study, we sought to explore whether osthole inhibits histamine-dependent itch via TRPV1. Our results showed that osthole clearly reduced the scratching behaviors induced by histamine. Osthole also suppressed the H1 and H4 receptor-mediated scratching behaviors. Furthermore, osthole decreased the response of DRG neurons to histamine, HTMT, VUF8430, and capsaicin by modulating the TRPV1 activity.

## Results

### Osthole-attenuated scratching behavior induced by histamine, HTMT, and VUF8430

**Figure 2:**
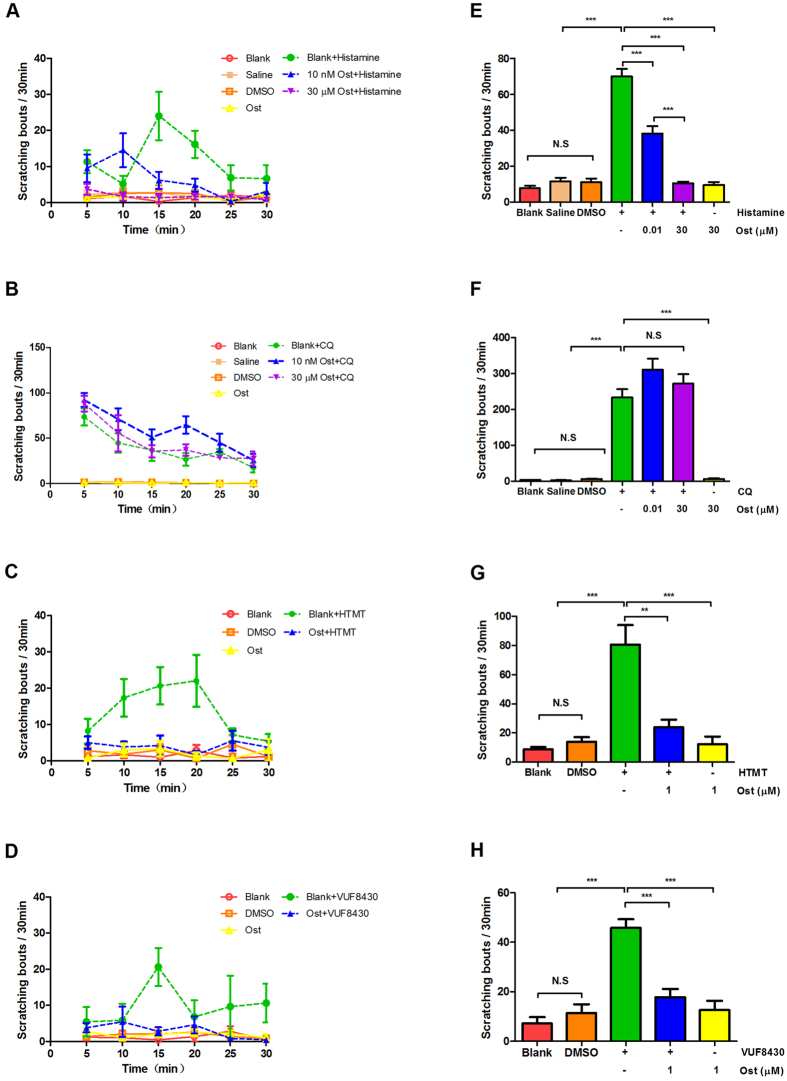
Osthole inhibited histamine and histamine agonist-induced scratching but not chloroquine in mice. Itch-related behaviors were determined by scratching bouts in 5-min as a bin during 30-minute periods following injection of vehicle and pruritogens 50 μl. (**A**,**E**) The animals were divided into seven groups; four of them were used as control (blank, saline, DMSO, osthole) drug groups including histamine, and different concentrations of osthole plus histamine were used to observe scratching behavior. The results showed that histamine can obviously induce the scratch behavior, and the different concentrations of osthole could significantly inhibit the scratch behavior of mice (*n* = 6). (**B**,**F**) As in the above experiment, the same control groups were adopted, but drug groups were replaced by CQ and the different concentration ostholes plus CQ. The results showed that the scratch behaviors by the CQ-induced were not inhibited by the different concentrations osthole (*n* = 4). (**C**,**G**) Osthole was able to inhibit the scratching of HTMT-induced (*n* = 6). (**D**,**H**) The scratch of VUF 8430-induced could also be inhibited by osthole (*n* = 5). The data are presented as mean ± SEM. (N.S., no significant, *p < 0.05, **p < 0.01, ***p < 0.001).

A previous study reported that a high dose of histamine can induce obvious scratching behavior^24^. To examine the anti-pruritic effect of the osthole on the histamine-dependent itch, a pretreatment by subcutaneous injection with osthole (10 nM, 30 μM, 50 μl/site) into the nape of the mouse neck was adopted. Histamine (100 μM, 50 μl/site) was injected into the same site 30 min later. The scratching bouts were counted for 30 minutes. The results showed that histamine obviously induced scratching behavior. By contrast, with the pretreatment of osthole, the histamine-induced (70 ± 4, *n* = 6) scratching bouts were significantly attenuated (10 nM osthole, 38 ± 4, paired *t*-test, *P* < 0.001; 30 μM osthole, 11 ± 1, paired *t*-test, *P* < 0.001) (Fig. 2A,E). Further, we examined the effects of osthole on the histamine-independent itch; however, osthole did not inhibit the chloroquine (CQ)-induced itch (Fig. 2B,F).

Similar to histamine, histamine H1 receptor agonist (HTMT) and histamine H4 receptor agonist (VUF8430)-induced scratching behaviors were both inhibited by osthole. The scratching bouts of HTMT-induced (0.1 μM, 50 μl/site) reduced from 81 ± 13 to 24 ± 5 (*n* = 6, paired *t*-test, *P* < 0.01) (Fig. 2C,G). As shown in Fig. 2D,H, the scratching bouts of VUF8430-induced (100 μM, 50 μl/site) decreased from 46 ± 4 to 18 ± 3 (*n* = 5, paired *t*-test, *P* < 0.001).

### Osthole inhibited the response of DRG neurons to histamine

**Figure 3:**
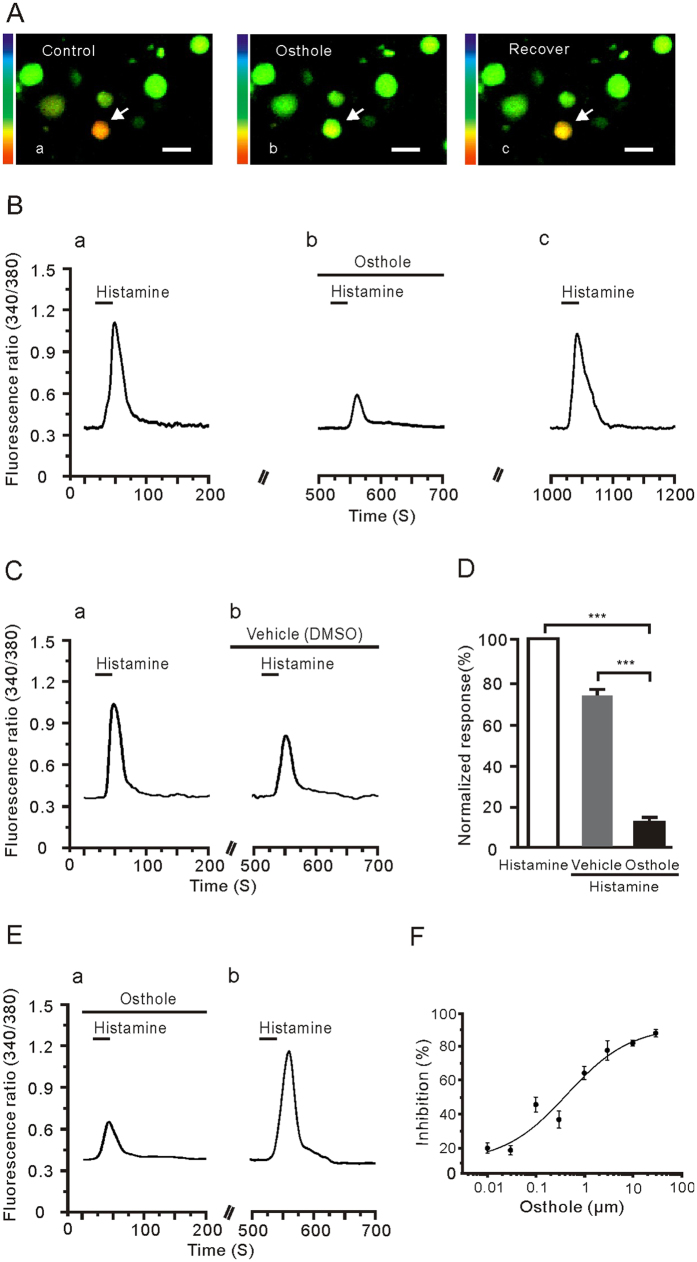
Osthole suppressed the histamine-induced response in DRG neurons. (**A**) Fluorescence image displays intracellular calcium flux induced by histamine. Arrows indicate the neurons’ response to 100 μM histamine (a), 1 μM osthole inhibition (b), and recover to histamine (c), respectively. (**B**) The representative trace showed that histamine-evoked calcium influx was obviously reduced by pretreatment with osthole (b) and recovered when histamine was applied again (c). (**C**) The repeated histamine stimulation also caused the reaction of the fluorescence ratio (340 nm/380 nm) to become small in the case of with the histamine together the control solution (1% DMSO) to perfuse. (**D**) The histogram of histamine-induced Ca^2+^ response indicated that the inhibitory response of osthole to histamine was much stronger than the desensitization caused by repeated use of histamine. Vehicle is a perfusion solution with 1% DMSO (***p < 0.001). (**E**) The inhibitory effect of osthole is not the desensitization of histamine repeated application. (**F**) The dose-response curve of osthole to inhibit the histamine-evoked calcium influx was displayed (IC_50_ ≈ 0.41 μM).

To determine whether osthole inhibited the histamine-evoked neuronal activation, we examined the histamine-evoked Ca^2+^ signal in acutely dissociated and cultured DRG neurons. Interestingly, the histamine-evoked calcium influx was notably, but not totally, inhibited by the pretreatment with osthole. After washing out, most of the histamine induced responses have been recovered (Fig. 3A,B). However, in the case of using 1% DMSO as a blank control, DRG neuron responses to hitamine were also reduced. This is probably because of the same neuron’s desensitization to repetitive histamine stimulation (Fig. 3C,D). In addition, the inhibition of osthole on histamine could be recovered well (Fig. 3E). Furthermore, the inhibition effect of osthole on histamine-evoked intracellular calcium influx was dose-dependent (IC_50_ ≈ 0.41 μM) (Fig. 3F). These data demonstrate that osthole has an inhibitory effect on histamine-evoked intracellular calcium flux on the DRG neurons.

### Osthole suppressed the response of DRG neurons to HTMT

**Figure 4:**
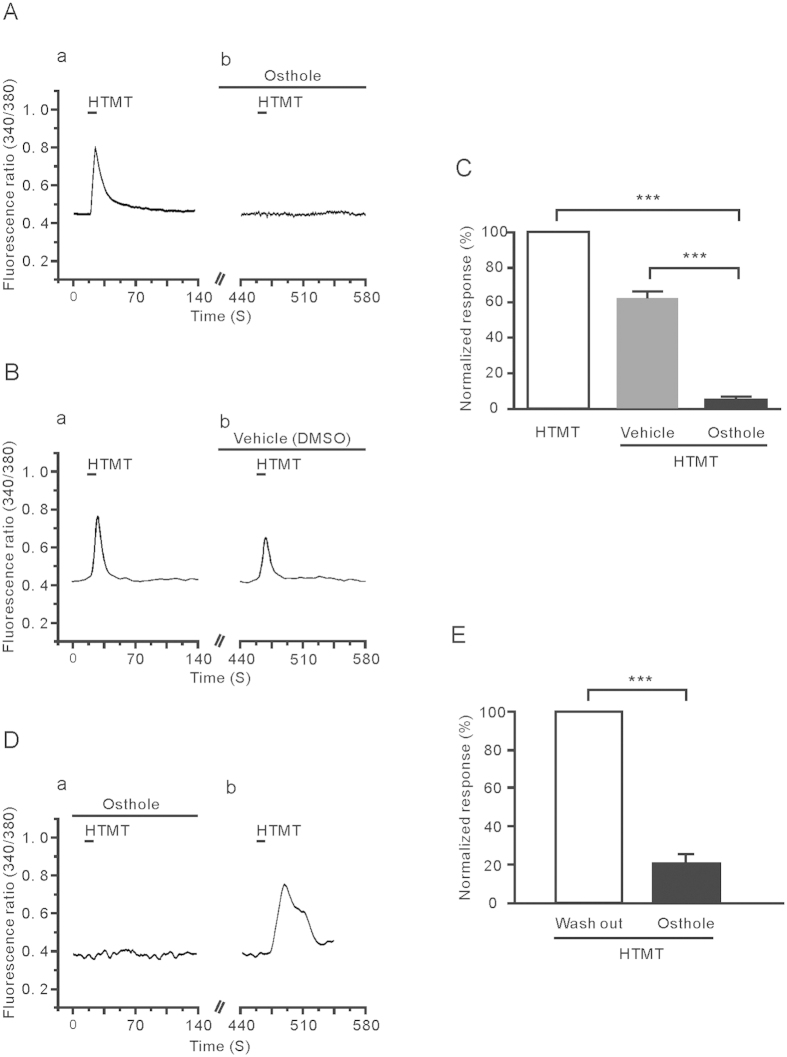
Osthole suppressed the HTMT-induced response in DRG neurons. (**A**) The representative trace showed that HTMT (1 μM) induced calcium influx (a); osthole (1 μM) almost completely blocked the response of HTMT induced (b). (**B**)The contrast solution including (1% DMSO) and HTMT application also showed a suppression phenomenon. (**C**) The inhibitory response of osthole to HTMT-induced was much stronger than the desensitization caused by repeated use of HTMT (***p < 0.001). (**D**) The inhibitory effect of osthole is not the desensitization of HTMT repeat application. (**E**) The normalized fluorescence intensity of osthole to inhibit HTMT-induced calcium influx is 22 ± 7% (*n* = 5, **p < 0.001).

The pruritogenic effect of histamine is mainly mediated by histamine H1 and H4 receptors^25^; therefore, to determine more precisely whether osthole blocks histamine-evoked intracellular calcium flux by different receptors, we examined the inhibition effect of osthole on HTMT-evoked calcium influx, which is a highly selective histamine H1 receptor agonist^26^. We found that the HTMT (1 μM)-evoked calcium influx was inhibited absolutely by osthole (1 μM) (Fig. 4A). After pre-perfusion of DRG neurons with osthole, the intensity of the neurons’ response to HTMT was obviously reduced compared with the vehicle (5 ± 1% VS 63 ± 4%, unpaired *t*-test, *P* < 0.001) (Fig. 4B,C). Further, by pre-perfusion of DRG neurons with osthole, HTMT-evoked calcium influx was obviously reduced. After washing, the application of HTMT again induced an obvious Ca^2+^ influx (*n* = 5, paired *t*-test, *P* < 0.001) (Fig. 4D,E).

### Osthole inhibited the response of DRG neurons to VUF8430

**Figure 5:**
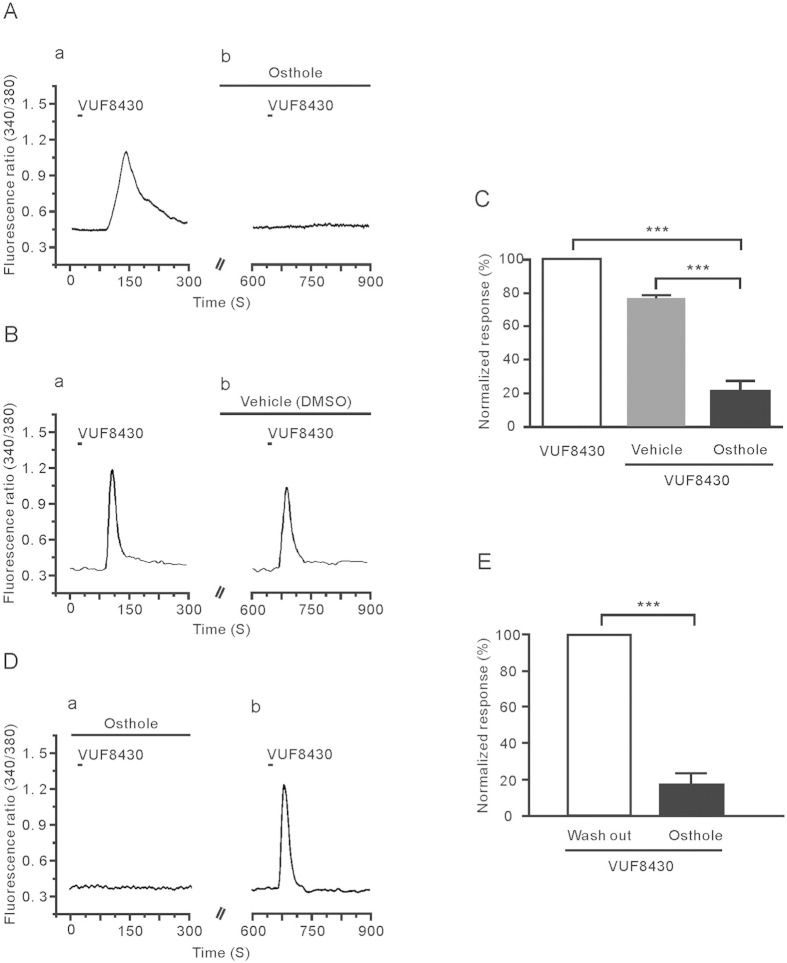
Osthole inhibited the VUF8430-induced response in DRG neurons. (**A**) The representative trace from calcium image showed 100 μM VUF8430-induced intracellular calcium influx (a); 1 μM osthole was applied before VUF8430 application again (b). (**B**)The representative trace showed the intracellular calcium influx that VUF8430 used alone and VUF8430 together with vehcle (1% DMSO). (**C**) The inhibitory response of osthole to VUF8430-induced was much stronger than the desensitization caused by repeated use of VUF8430 (***p < 0.001). (**D**) The inhibitory effect of osthole is not the desensitization of VUF8430 repeated application. (**E**) The normalized fluorescence intensity of osthole to inhibit VUF8430 induced calcium influx is 17 ± 6% (*n* = 13, **p < 0.001). The inhibitory effect reached about 83%.

Does osthole have a characteristic of broad spectrum to block histamine-mediated response or only have a specific inhibitory effect on H1 receptor-mediated response? To clarify this hypothesis, we tested the effect of osthole on H4 receptor agonist-evoked intracellular calcium flux. As shown in Fig. 5, similar to histamine, a highly selective histamine H4 receptor agonist, VUF8430, evoked a remarkable Ca^2+^ influx in the DRG neuron. By pre-perfusion of DRG neurons with osthole, VUF8430-induced Ca^2+^ signal was reduced compare to the vehicle (21 ± 6% vs 75 ± 3%, unpaired *t*-test, *P* < 0.001) (Fig. 5A–C). To consider the desensitization of VUF8430-induced response, we repeated the same test as before, but the order of osthole application was changed, as shown in Fig. 5D. The result indicated that the 83 ± 6% of VUF8430-induced Ca^2+^ signal was increased after washout (*n* = 13, paired *t*-test, *P* < 0.001) (Fig. 5E). In these DRG neurons’ response to VUF8430, 23% of them were totally inhibited by osthole; the other neurons were partly inhibited. These results indicate that osthole had an inhibitory effect on the H4 receptor-mediated responses.

### Osthole inhibited the DRG neurons’ response to histamine and capsaicin

**Figure 6:**
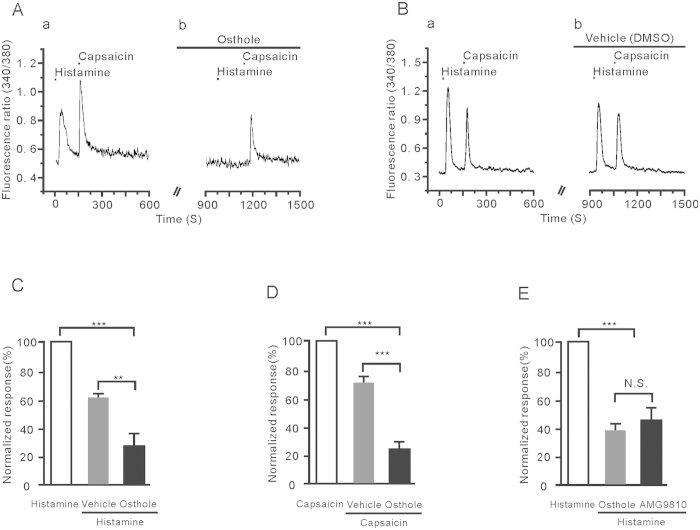
Osthole inhibited both histamine and capsaicin-induced calcium influx on the same DRG neurons. (**A**) The traces of calcium influx induced by 100 μM histamine and 1 μM capsaicin (a) were inhibited by 1 μM osthole (b). (**B**) The response curves of histamine and capsaicine were added respectively in the normal perfusion and the vehicle (1% DMSO) solution. (**C**,**D**) The normalized fluorescence intensities of osthole to inhibit histamine (**C**) and capsaicine (**D**)-induced calcium influx are 29 ± 6% (n = 16, **p < 0.01) and 23 ± 16% (n = 16, ***p < 0.001), respectively. (**E**) Osthole (IC50 ≈ 0.41 μM) and AMG9810 (IC50 ≈ 0.1 μM) were able to significantly inhibit the response induced by histamine. However, the inhibitory effects between osthole and AMG9810 to histamine-induced responses were not significantly different.

Studies have suggested that TRPV1 mediates the histamine signal transduction in primary sensory neurons^27^,^28^. To further investigate whether the TRPV1 is involved in the inhibitory effects of osthole on histamine-evoked response, we pretreated the neurons with osthole, and then applied histamine and capsaicin to the same neurons. As shown in Fig. 6A, histamine and capsaicin (1 μM) both evoked Ca^2+^ influx in the same neurons. After pre-perfusion of osthole, the following addition of histamine and capsaicin-induced response were both reduced. Compared with the first histamine and capsaicin treatment, histamine-induced response decreased to 29 ± 6%, capsaicin-induced response decreased to 23 ± 16% (Fig. 6C,D). These results suggest that osthole may be a modulator of TRPV1 to inhibit histamine-induced Ca^2+^ influx in DRG neurons. Interesting, we found that the AMG9810, a potent TRPV1 antagonist, has a similar inhibitory effect on histamine compare to osthole (Fig. 6E).

### Osthole directly modulated the response of DRG neurons to capsaicin

**Figure 7:**
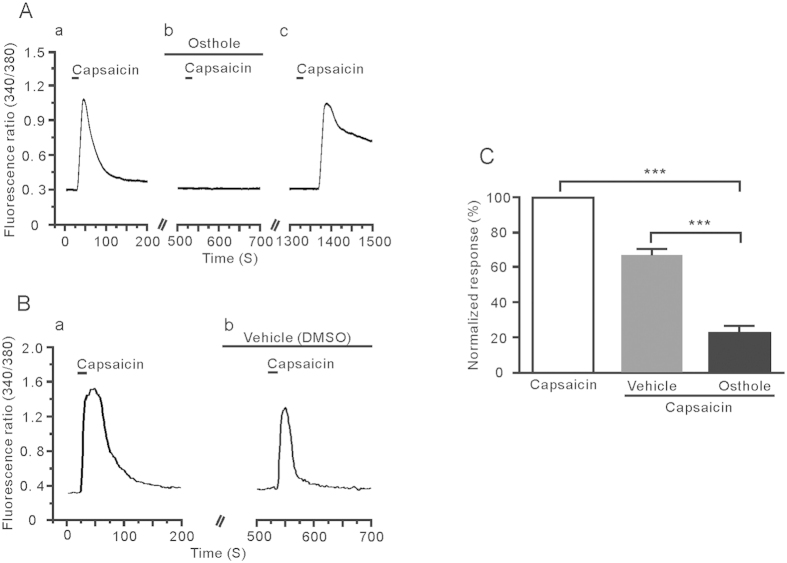
Osthole directly modulates TRPV1 activity. (**A**) Osthole could completely block the response induced by capsaicin. After 10-minutes washout, the response to capsaicin-induced recovered. (**B**) The representative trace showed the intracellular calcium influx that capsaicine used alone and capsaicine together with vehcle (1% DMSO). (**C**) The normalized fluorescence intensities of osthole to inhibit capsaicine -induced calcium influx.

It has been proven that histamine H1 receptor-induced itch signal transduction needs TRPV1 activation^29^, and we observed that osthole may regulate TRPV1 Ca^2+^ influx of histamine-induced response in DRG neurons. To determine whether osthole has a direct modulating effect on TRPV1, we investigated the effect of osthole on TRPV1. As we speculated, 1 μM osthole pretreatment evidently decreased the capsaicin-evoked intracellular calcium levels. As the previous controlled trail, capsaicin-induced Ca^2+^ signal was reduced when osthole was pre-perfused (23 ± 4% vs 67 ± 4%, unpaired *t-*test, *P* < 0.001) (Fig. 7A–C). Furthermore, since pharmacological desensitization of TRPV1 is always a disturbing artifact for judging the osthole effect, we washed out the capsaicin-induced response for 5 to 10 min, to prevent interfering of TRPV1 desensitization. As shown in Fig. 7A, capsaicin-induced Ca^2+^ influx was totally blocked by the osthole, but, after a 10-min washout with normal solution, the response to capsaicin recovered. These results suggest that osthole directly modulate TRPV1 activity in the DRG neurons.

### Osthole suppressed the capsaicin-induced inward current

**Figure 8:**
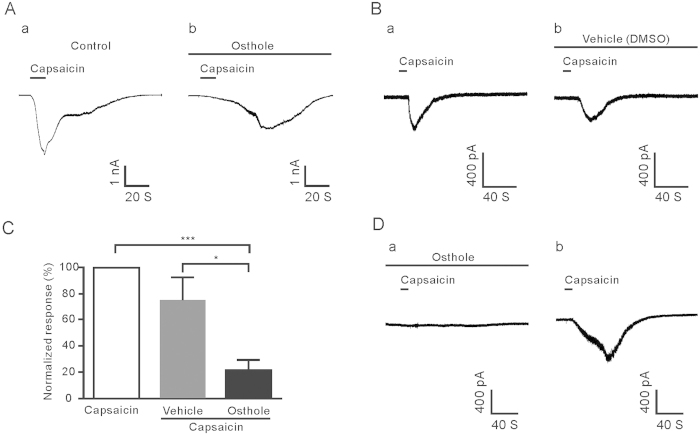
Osthole suppressed the inward current of capsaicin-induced. (**A**) 1 μM capsaicin evoked inward current from whole-cell recording (a) was inhibited by 1 μM osthole (b). (**B**) The representative trace showed capsaicin-induced inward current in the presence of the normal and vehicle (1% DMSO) solution to perfuse. (**C**) The normalized current intensities of osthole to inhibit capsaicine -induced inward current. (**D**) Osthole almost totally blocked the inward current of 1 μM capsaicin–induced, but the response capsaicin-induced recovered after 5-minutes washout.

To further investigate how osthole modulates the TRPV1 channel, we used a whole-cell voltage clamp recording to examine the inhibitory effect of osthole on TRPV1 current in dissociated and cultured small (<25 μm) DRG neurons. These cells were held at −60 mV. Application of capsaicin (1 μM) alone evoked an inward current; however, with the pretreatment of osthole (1 μM), capsaicin-induced current intensity was reduced compare to the vehicle (22 ± 8% vs 74 ± 20%, unpaired *t-*test, *P* < 0.05) (Fig. 8C). As shown in the sample in Fig. 8A, the peak current by 1 μM capsaicin-induced was reduced from 2927 pA to 1718 pA with the pretreatment of osthole. To distinguish the inhibitory effect of osthole without desensitization of TRPV1, we first pretreated with osthole and found that the capsaicin-induced inward current was completely blocked. Then, we rinsed the recording neurons for 5 minutes with normal solution and observed that the inward current recovered after the addition of capsaicin again (Fig. 8D).

## Discussion

Itch (pruritus), including chronic and acute itch, is a disease that seriously affects the quality of life. A survey in France found that 28.7% of individuals had chronic itch; a survey from Germany^3^ found that 16.5% of individuals reported itchy skin. Although many methods are used as clinical treatment for chronic and acute itch, their efficacies are limited. It is necessary to develop a new, efficacious, antipruritus medication. Studies have found that the ethanol extract of *Cnidium monnieri Fructus* has an anti-inflammatory effect on DNFB-induced contact dermatitis. Furthermore, ethanol extract of *Cnidium monnieri Fructus* also has an antipruritus effect for the 5-HT, compound 48/80, and SP-induced itch^22^,^23^,^30^,^31^. Administration of osthole, however, did not inhibit SP-induced scratching. In contrast, osthole showed an inhibitory effect on compound 48/80-induced scratching, which suggests that osthole has an inhibitory effect on histamine-dependent itch. Our results indicate that osthole clearly reduced histamine-induced scratching behavior.

It is now known that four receptors (H1–H4 receptors) mediate histamine action. Histamine H1 and H4 receptors play a key role in histamine-induced itch signal transduction in peripherals. Neither histamine H1 receptor antagonist nor H4 receptor antagonist can completely block histamine-induced scratching behavior. The histamine-induced scratching behavior was almost blocked only when we used both histamine H1 and H4 receptor antagonists^32^. Second, mepyramine (H1 receptors antagonist) could not reduce scratching behavior induced by clobenpropit (H4 receptor agonist), and HTMT-induced scratching behavior also could not be reduced by thioperamide (H3/H4 antagonist)^24^. These reports suggest that histamine H1 and H4 receptors are co-involved in the pathway to transmit the itch signal to the center system. In the present study, we showed that osthole could obviously reduce both histamine H1 and H4 receptor agonist-induced scratching behaviors. This study indicated that osthole may not be a selective agent of H1 or H4 receptor directly. Osthole plays a partial role via the conjunction of H1 and H4 receptors to prevent their downstream signal transduction.

The histamine H1 receptor is coupled with Gαq proteins. When the H1 receptor was activated, the Gαq downstream signal pathway induced TRPV1 to open and excited the neurons to transmit the itch signal^11^,^33^. In our previous studies, TRPV1 was also the downstream ionic channel of histamine H4 receptor^34^. Therefore, we speculate that osthole inhibits histamine-dependent itch by modulating the TRPV1 activity. Indeed, we found that osthole inhibits an increase in [Ca^2+^]_i_ and the inward current of the DRG neurons by capsaicin inducement. These results indicate that TRPV1 plays an important role in osthole inhibition to capsaicin-induced responses. Surprisingly, a high concentration of osthole was able to directly induce an increase of [Ca^2+^]_i_ in the DRG neurons, but a low concentration of osthole did not. Therefore, we speculate that osthole under high concentration may play a role in facilitating TRPV1 desensitization similar to *furanocoumarin imperatorin*, a novel class of TRPV1 partial agonists that facilitate TRPV1 desensitization and that potentiate acid activation of TRPV1^35^. Several lines of data suggest that TRPV1 may function as a molecular integrator in histamine-independent itch. Trypsin-induced itch was decreased by genetically deleted or blocked TRPV1^13^. IL-31-induced scratching behavior was significantly attenuated in TRPV1 KO mice^36^. TRPV1 also has a similar role in pain regulation^37^. Because osthole is closely related to the function of TRPV1, osthole may also be used to treat pain disease related to TRPV1, such as postherpetic neuralgia, trigeminal neuralgia, and osteoarthritis^38^,^39^. Our findings support the hypothesis that the sensation of pain or itch is dependent on the type of neurons, not on the ion channels^40^.

However, many other forms of itch—which induce robust scratching behaviors and signals via distinct histamine-independent molecular pathways—are insensitive to the treatment of anti-histamine, for instance: cowhage, chloroquine, Ser-Leu-Ile-Gly-Arg-Leu (SLIGRL), β-alanine, bovine adrenal medulla peptide (BAM) 8–22, thymic stromal lymphopoietic protein (TSLP)^41^,^42^,^43^. But, the chloroquine-induced scratching behaviors in mice were not inhibited by pretrement of osthole. It indicates that the antipruritic effect of osthole mainly depends on histamine-dependent pathway.

In summary, Osthole is an inhibitor of histamine-induced scratching behavior, at least in part to suppress the itching. In peripheral sensory neurons, TRPV1 is involved in the osthole inhibition of the histamine-dependent itch. Although these findings are preliminary, this study opens a window to explore and examine osthole as a novel anti-pruritic treatment for histamine-dependent itch.

## Materials and Methods

### Animals

C57BL/6 male mice (8–10 weeks) were used for behavioral testing (Experimental Animal Center, Nanjing University of Chinese Medicine, Nanjing, China). Mice were housed in a temperature-controlled animal room (22 ± 2 °C) under a 12-h light/dark cycle, with free access to food and water. The study was performed in accordance with relevant guidelines and regulations of the Institutional Animal Care and Use Committee of the Nanjing University of Chinese Medicine. All experimental protocols were approved by the International Association for the Study of Pain.

### Drugs

Histamine dihydrochloride, chloroquine diphosphate salt, VUF 8430 dihydrobromide, histamine trifluoromethyl toluidide (HTMT), osthole, capsaicin, and AMG9810 were obtained from the Sigma-Aldrich Corp. (St. Louis, MO, USA). With the exception of histamine and chloroquine, which were dissolved in water, all the other drugs were dissolved in DMSO. When the drugs were used in the behavior experiments, the drugs were diluted in saline, then the calcium imaging and the electrophysiological experiments, all the drugs were diluted in normal perfusion solution, the final concentration of DMSO or water did not exceed 0.5%.

### Behavioral Assays

The rostral part of mice neck was clipped and depilated with electric hair clippers 24 h before starting the experiments. Mice were placed in a box (4.5 × 4.5 × 7 inch) for approximately 30 min for acclimatisation before each experiment. All drugs were injected subdermally via a 30G needle into the rostal part of the neck. Immediately after the injection of the drugs, mice were recorded on video for 30 minutes and the number of scratch bouts counted at 5-min intervals by an investigator blinded to treatment. The four groups in the experiments included a blank group, saline group, solvent group (DMSO 0.5% in saline), and osthole group. For excluding osthole-induced scratching behaviors, we also recorded the behaviors on video for 30 minutes followed injection of osthole. In the inhibited experiments, osthole (0.01, 1, 30 μM) was administered by subcutaneous injection 30 min before the subcutaneous injection of histamine (100 μM), chloroquine (8 mM), HTMT (0.1 μM), and VUF8430 (100 μM). All drugs were injected in a volume of 50 μl. One scratch response was defined as a lifting of the hind limb towards the injection site. All behavioral experiments were conducted with the observers blinded to treatments.

### Culture of dissociated DRG neurons

Acutely dissociated DRG neurons from adult mice (4–6 weeks old) were collected in cold DH10 (90% Dulbecco’s modified Eagle medium [DMEM]/F-12, 10% fetal bovine serum [FBS], Penicillin [100 U/ml] and streptomycin [100 μg/ml]) (Gibco, USA), and treated with enzyme solution (dispase [5 mg/ml], collagenase type I [1 mg/ml]) in Hanks’ Balanced Salt Solution (HBSS) without Ca^2+^ and Mg^2+^ (Gibco, USA) at 37 °C for 30 min. Dissociated cell suspensions were filtered through a 100-μm cell strainer (BD, Franklin Lakes, NJ, USA). After trituration and centrifugation at 1200 rpm for 5 min, the cells were resuspended in DH10, and nerve growth factor was added (50 ng/mL, Millipore, Billerica, MA, USA). Suspended cells in DH10 solution were plated on glass coverslips coated with poly-D-lysine (0.5 mg/ml, sigma) and laminin (10 μg/ml, Invitrogen), and cultured in an incubator (95% O_2_ and 5% CO_2_) at 37 °C.

### Calcium imaging

Dorsal root ganglia were dissociated cultured from 4–6-week-old mice for 16–18 h. For Ca^2+^ imaging experiments, the cells were loaded with Fura-2-acetomethoxyl ester (molecular Probes, Eugene, OR, USA) in HBSS solution for 30 minutes in the dark at room temperature. After washing 3 times, the glass coverslips were placed into a chamber and perfused with normal solution. A high-speed, continuously scanning, monochromatic light source (Polychrome V, Till Photonics, Gräfeling, Germany) was used for excitation at 340 and 380 nm, enabling us to detect changes in intracellular free calcium concentration. Cells were bothed in the normal solution (in mM): 140 NaCl, 5 KCl, 10 HEPES, 2 CaCl_2_, 2 MgCl_2_, 10 Glucose, and pH 7.4 with NaOH to adjust. A baseline reading was taken for 20 s before applying histamine, HTMT, VUF8430, and capsaicin to DRG neurons.

### Whole-cell patch clamp recording

In voltage clamp recordings, currents were recorded with an Axon 700B amplifier and the pCLAMP 10.1 software package (Axon Instruments). Cells were bathed in normal solution (in mM): 140 NaCl, 4 KCl, 2 CaCl_2_, 2 MgCl_2_, 10 HEPES, 5 Glucose, pH 7.4 in NaOH to adjust. Pipette resistance ranged from 2 to 5 MΩ. The internal solution (in mM) was 35 KCl, 3 MgATP, 0.5 Na_2_ATP, 1.1 CaCl_2_, 2 EGTA, 5 Glucose, pH 7.4 in KOH to adjust, and osmolarity was adjusted to 300 mosM in sucrose. Capsaicin was stored at −20 °C and diluted to 1 μM in the extracellular solution. Electrodes were pulled (Sutter, model P-97) from borosilicate glass (Sutter). All experiments were performed at room temperature.

### Data analysis

All data were expressed as the mean ± SEM. Statistically significant differences between the vehicle and osthole treatment were assessed by a one-way ANOVA. A comparison of only two groups was done by means of a *t*-test. N.S, no significant. *p < 0.05, **p < 0.01, and ***p < 0.001 represent statistically significant differences.

## Additional Information

**How to cite this article**: Yang, N.-N. *et al.* Osthole inhibits histamine-dependent itch via modulating TRPV1 activity. *Sci. Rep.* **6**, 25657; doi: 10.1038/srep25657 (2016).

## Figures and Tables

**Figure 1:**
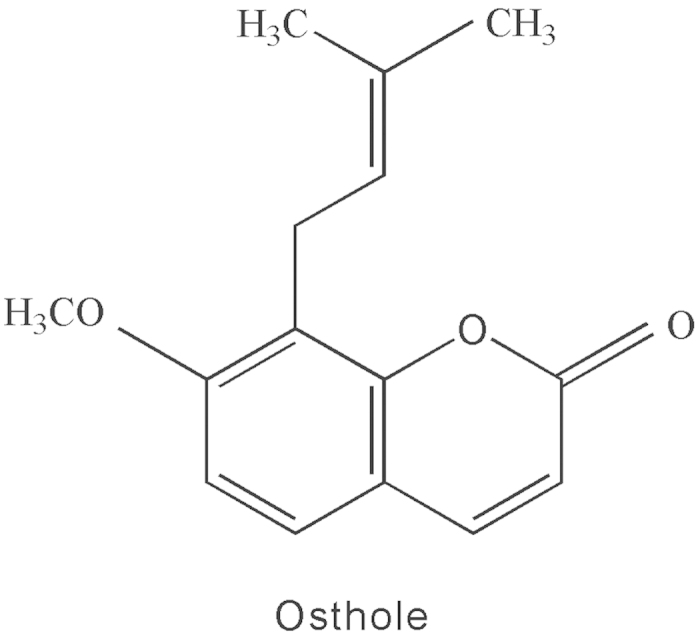
Chemical structure of osthole.
